# A volunteer-supported walking programme to improve physical function in older people (the POWER Study): study protocol for a randomised controlled trial

**DOI:** 10.1186/s12877-020-01988-9

**Published:** 2021-01-13

**Authors:** Nina Grede, Anja Rieckert, Julia Muth, Joana Steinbuck, Sabine Weissbach, Annika Schneider, Beate Weber-Schicker, Ellen Freiberger, Nikita Jegan, Norbert Donner-Banzhoff, Andreas Sönnichsen

**Affiliations:** 1 Department of General Practice/Family Medicine, Philipps-University of Marburg, Marburg, Germany; 2 Department of Human Medicine, Institute of General Practice and Family Medicine, Faculty of Health, Witten/Herdecke University, Witten, Germany; 3 Institute for Biomedicine of Aging, Friedrich-Alexander University Erlangen-Nürnberg, Nuremberg, Germany; 4 Department of General Practice and Family Medicine, Center for Public Health, Medical University of Vienna, Vienna, Austria; 5 Division of Population Health, Health Services Research and Primary Care, School of Health Sciences, University of Manchester, Manchester, UK

**Keywords:** Healthy ageing, Older people, Exercise, Physical activity, Quality of life, Peer-support, Walking

## Abstract

**Background:**

Currently 21% of the German population is older than 65 years. Above this age, the risk of suffering from chronic disease and mental disorders increases rapidly. Therefore, physical inactivity is one of the most important public health concerns among older people. To address this issue, we have conceptualised and evaluated a simple and low-threshold intervention, which requires only minimal demand on the participants, targeting older people with inadequate activity levels. The aim of the POWER Study is to investigate whether volunteer-supported outdoor-walking improves physical function and quality of life in older people.

**Methods/design:**

In a randomised, controlled interventional superiority-trial, individuals older than 65 years of age living in the community or nursing homes will be randomised into two groups. The study will be conducted in two study centres with assessments at baseline, 6 and 12 months. The intervention group will participate in a supported physical activity intervention for 6 months. An assigned volunteer will visit them three times a week for an outdoor walk between 30 and 50 min, or equivalent indoor activity. Persons in the control group will be invited to two lectures covering topics related to health. Primary endpoint is the physical function measured by the Short Physical Performance Battery (SPPB) at baseline, after 6 and 12 months. Secondary and safety endpoints will be quality of life (EQ. 5D), fear of falling (Falls Efficacy Scale), physical activity (activity diary), cognitive executive function (clock drawing test), falls requiring medical attention, hospitalisation and death. Primary analysis will be carried out by intention to treat.

**Discussion:**

We expect the intervention to improve the overall health status of the participants in a wide range of health-related outcomes. If effectiveness can be shown, the intervention will close an important gap in current services for older people. We will disseminate our experiences and results in the form of informational documents (training manual) to allow municipalities and health care organisations to implement a similar intervention.

**Trial registration:**

The trial was registered on 31 Aug 2018; German Clinical Trials Register (www.germanctr.de), Deutsches Register Klinischer Studien: DRKS00015188.

**Supplementary Information:**

The online version contains supplementary material available at 10.1186/s12877-020-01988-9.

## Background

Currently about 81 million people are living in Germany, of which 21% are older than 65 years [1]. While the total population will presumably increase to 82 million in the year 2040, the proportion of people older than 65 years will increase to 26% [2]. After the age of 65, the risk of suffering from 2 or more chronic diseases, metabolic conditions as well as the risk of falls increases rapidly [3]. The risk of falling also increases with age. More than 10% of all individuals between the age of 65 and 80 years have fallen more than once during the year prior to the interview [4, 5]. The more chronic diseases somebody experiences, the higher is the probability for mental health disorders (e.g. depression) [4]. A German population-based study showed that the odds for suffering from clinically depression are almost twice as high in people with four chronic diseases as compared to those with two [4].

To prevent the development or exacerbation of chronic diseases in older individuals, it is essential to maintain regular physical activity. While the World Health Organization (WHO) recommends at least 2.5 h of moderate physical activity per week [6], 73% of German women and 67% of German men above the age of 65 years do not meet this recommendation [7]. Therefore, physical inactivity is one of the most important public health concerns, especially in older people. Despite an increase in age-targeted activities offered by public health and community organisations, the proportion of people above 70 years who are sufficiently active is still below 14% [8]. Therefore, engaging means to stimulate sports and other physical activity are required.

To our knowledge, so far only a few studies examined the effects of a walking intervention on community dwelling individuals. A British mixed-methods study examined a sample of people between 60 and 75 years who were recruited in primary care practices [9]. This complex intervention included feedback by pedo- and accelerometers, scheduled practice nurse consultations and activity diaries to establish regular self-paced walking. There was an improvement in the primary outcome “daily step-counts” and the participants reported gains in health and wellbeing in the qualitative component of the study [9]. However, the quantitative component lacked these and other health related measures to back up the qualitative findings (e.g. physical function, quality of life). Other major limitations of the study are the need for qualified staff and expensive technological equipment [9]. An Australian study on community dwelling individuals aged 65+ years evaluated a self-paced 48 week walking programme that involved mailed printed manuals and telephone coaching [10]. The intervention successfully led to an increase in activity and physical function as measured by the Short Physical Performance Battery. In addition, the sample was relatively fit and lacked older individuals with mild and moderate limitations [10]. Another pilot study showed that a combination of initial motivation by a peer volunteer and subsequent activity such as walking in groups, yoga or dancing led to behavioural changes in older people and improved physical activity [11].

Taken together, these studies show that interventions aimed at promoting regular walking improve older persons’ health. However, the interventions were complex and required professional input. Moreover, individuals who already had physical, sensory and/or cognitive limitations were not or not sufficiently included in the community dwelling studies.

The aim of the POWER Study is to investigate whether volunteer-supported outdoor-walking improves the physical function and quality of life of older people. A further aim is the implementation of the intervention in existing aid structures (e.g. regional health department). The POWER Study design has been conceptualised and piloted [12] as a simple and low-threshold intervention that is targeted at older people with inadequate activity levels, already present minor restrictions in physical and mental functioning preventing them from self-paced physical activity, and a lack of support.

## Methods/design

We are carrying out a randomised, controlled interventional superiority-trial with two parallel groups with physical function as primary endpoint to examine whether volunteer-supported walking interventions for older people improve physical health. Measurements will be done at baseline (T0), at 6 months (T1) and at 12 months (T2) (see Table 1).

**Table 1:** Measures use for the assessments

Outcome Measure	Operationalisation (Type of assessment)	Times of assessment		
		T0	T1	T2
**Participant**				
Sociodemographic Data	Age, sex, level of care, family status, education level, ethnicity	X		
Physical Function	Short Physical Performance Battery	X	X	X
Quality of Life	EQ-5D-5L	X	X	X
Fear of Falling	Falls Efficacy Scale	X	X	X
Frailty	Clinical Frailty Scale	X	X	X
Cognitive Changes	Clock-drawing-test	X	X	X
Cognitive Impairment	Mini Mental Status Examination (MMSE)	X		
Walk Assessment	Number and duration of walks, assistance, type of assistance		X	X
Falls	Type and place of treatment		X	X
Treated Falls	Date, ICD Code, falls		X	X
Hospitalisation	Date of hospital admission and discharge, duration of hospitalisation, reason for hospitalisation (ICD-10-Code)		X	X
Death	Date of death, Reason for death (ICD-10-Code)		X	X
**Volunteer**				
Sociodemographic Data	Age, sex, job, family status, education level, ethnicity, volunteering history			

### Aims

Our research questions are:

1. Compared to controls, does volunteer-supported walking improve physical function and quality of life as well as lower fear of falling? Furthermore, improves supported walking the cognitive executive function and the general physical activity? [Effectiveness]
2. Compared to controls, does volunteer-support walking increase the rate of falls needing medical attention, rate of admissions to hospital, duration of hospitalisations and mortality? [Safety]
3. Compared to controls, does supported walking have an impact on the frequency and duration of hospitalisations and/or mortality? [effectiveness, safety]

Finally, we explore whether the intervention can be successfully implemented into existing community services.

### Setting

A cohort of participants 65 years and older from North Rhine-Westphalia (Germany) and Hesse (Germany) will be recruited and followed up for 12 months. Participants will be recruited from nursing homes and from the community setting including general practitioners and home care nursing services.

### Eligibility

Eligibility criteria will be assessed at baseline.

Inclusion criteria:

- People aged ≥ 65 years
- Reduced physical function (Short Physical Performance Battery Score (SPPB [13];)
  < 9 at baseline)
- Not confident to go for a walk on their own

Exclusion criteria:

- Not giving informed consent
- Mini-mental status examination (MMSE) score at baseline < 18 [14]
- Insufficient physical function to allow for volunteer-supported walking with adequate safety (SPPB at baseline ≤2 in nursing homes and ≤3 in the community setting)
- Excellent physical function making it improbable that participants will benefit from the intervention (SPPB-Score ≥10)
- Permanently bedridden
- Can only be mobilised in a wheelchair
- Regular physical activity levels at least equivalent to the intervention (> 150 min/week)
- Life expectancy of less than 6 months
- Other foreseeable inability to take part in the intervention for 6 months
- Known alcohol or drug addiction or active psychosis during the last 12 months
- Another person of the same household participating in the study

Individuals using a walking frame, a cane or a wheeled walker will be included, as well as those suffering from Parkinson’s disease or mild cognitive impairment (MMSE ≥18). We expect all of these to benefit from the intervention, and we presume that volunteers will be able to cope with these disabilities or restrictions.

Eligibility criteria will be communicated to recruiting organisations, such as nursing homes or primary care practices. The final decision regarding eligibility will be made at the baseline examination, since some in- or exclusion criteria are formally assessed on this occasion, such as cognitive or physical function.

### Volunteers

Cooperating partner organisations (e.g. volunteer agency) will recruit volunteers. Advertisements in local newspapers, internet forums and bulletin boards (e.g. of universities and schools) will also be used. The minimum age will be 16 years, which is the minimum age for helpers in the federal volunteering service (Bundesfreiwilligendienst), and the possession of a mobile phone. Sufficient proficiency in German, sufficient self-reported physical fitness to be able to assist and availability for at least 6 months will be required. The number of participants assigned to one volunteer will be based on his/her time available and the physical condition of the participants.

The training is scheduled to take 6 h and includes instructions with regards to assisting older people (e.g. handling of tools like wheeled walker) and the documentation of the walks. Afterwards, volunteers will be assigned to the participants. Preferences will be taken into account (e.g. assistance a female person, participant living near home). They will be instructed to schedule the appointments with the participants themselves.

### Recruitment of participants

General practitioners, home care nursing services and nursing homes will be approached to support recruitment of patients and residents for the study. If interested, their staff will identify and screen candidate participants for eligibility. In the nursing home setting, all eligible residents will be invited to participate if they meet the inclusion criteria. General practitioners and nursing services will be asked to recruit each participant meeting the inclusion criteria until the recruitment goal is met. We will also approach potential participants through local newspapers, agencies, leaflets (e.g. in shops and pharmacies) and by word of mouth.

### Randomisation

Participants will be randomly assigned to either control or experimental group with a 1:1 allocation as per computer-generated randomisation schedule stratified by setting (community [primary care practice, nursing services] or nursing home). Participants will be randomly allocated via a telephone service provided by the Clinical Trial Centre at the University of Marburg (Koordinierungszentrum für klinische Studien [KKS]).

Research assistants will perform the data collection and examinations at baseline (T0) after 6 months of the intervention (T1) and after 12 months of follow up (T2). They will visit each participant either at their home or care home.

### Blinding

Blinding of participants is not possible due to the type of intervention. For practical reasons, study personnel cannot be blinded either, since at follow-up examinations participants are likely to communicate the study arm they have been allocated to. Moreover, assessments at baseline and follow-up of all participants, management of the intervention and other project tasks will be completed by the same members of the research team. A separation of tasks will not be possible. However, since randomisation will occur only after the baseline examination has been conducted, full concealment of allocation will be assured. In order to keep data collection at follow-up visits as unbiased and consistent as possible, a highly standardised protocol has been developed and will be implemented accordingly.

The study statistician will be blinded to participants’ allocation by being provided only pseudonymised data sets without explicit group allocation (only numbers will be used without revealing which was the intervention or control group).

### Study procedure and intervention

After baseline assessments (T0) and randomisation, individuals in the intervention group will participate in a supported physical activity intervention for 6 months. They will be visited by an assigned volunteer three times a week to go for a walk outdoors. The duration of the walk will depend on the participant’s physical ability. The aim is to gradually increase walking duration to three times 50 min to meet the WHO recommendation of 150 min per week [6]. In case of bad weather, the activity can take place indoors and will consist of exercises for balance and strength based on a programme of the federal centre for health education [15]. Each participant and his/her volunteer together will keep an activity diary, noting the date, time, duration and type of each activation exercise (outdoors or indoors). Events relevant for safety, e.g. falls or injuries, will also be recorded. Furthermore, after each walk, the subjectively experienced physical strain of the walk will document by a visual analogue scale.

Participants in the control group are invited to two lectures covering topics related healthy ageing during the 6 months of the intervention. The presentations will be in an easy to understand and entertaining manner and address health related topics for older people, e.g. nutrition. After 6 months of intervention the post intervention examination will take place (T1). We will evaluate the effect of the intervention in comparison with the control group. Study participants in both groups will be followed for another 6 months until the 12 months final examination (T2). During this period, the study centres will neither provide nor coordinate any walking service. However, we expect the collaborating partners and other community services to continue providing support for regular walking as initiated during the first 6 months of the study.

**Fig. 1:**
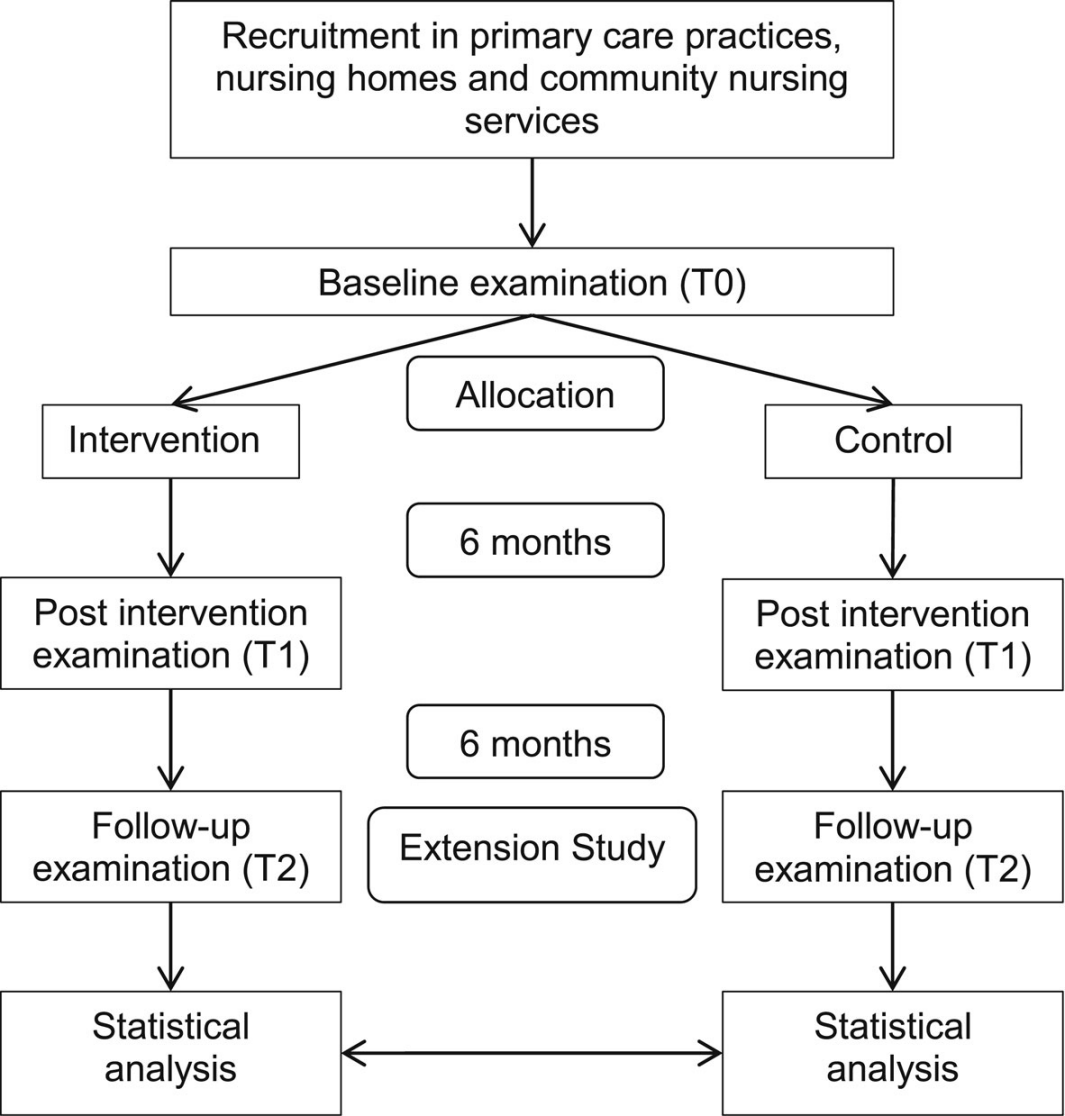
Study Flowchart

The evaluation of the extended period at T2 has two main objectives. First, to prolong participants’ follow-up and to assess long-term effects of regular walking (up to 12 months) and second, to evaluate potential sustainability and dissemination of the study intervention after cessation of support from the study centres (Fig. 1).

### Measurements

#### Demographic data (T0)

To characterise the participants, we will collect the following sociodemographic data: age, sex, body weight and height, educational level, family status, ethnicity and care level if applicable.

#### Mini mental status examination (T0)

For detecting cognitive impairment, the Mini Mental Status Examination (MMSE) [14] is used to check eligibility and will only be performed at baseline [16]. It is a widely utilised tool designed as an interview with the participant assessing central cognitive functions.

#### Short physical performance battery (T0, T1, T2)

The Short Physical Performance Battery (SPPB) includes several supervised activity tests rating balance, gait speed and the ability to get up from a chair in form of an external assessment [13].

#### Quality of life (T0, T1, T2)

Quality of life will be assessed using the validated German version of the EQ-5D-5L questionnaire, developed by the EuroQol Group [17]. This tool provides a simple, generic measure of health-related quality of life for clinical and economic appraisal.

#### Falls efficacy scale (to, T1, T2)

We will use the Falls Efficacy Scale *(*FES-I) questionnaire [18, 19], which allows for a convenient measure of falls related self-efficacy with excellent reliability [20], validity [21] and sensitivity to change [22].

#### Frailty (T0, T1, T2)

Frailty will be measured with the Clinical Frailty Scale (CFS) [23], which is a quick and valid subjective assessment tool of frailty.

#### Clock-drawing-test (to, T1, T2)

The Clock-drawing-test (CDT) is a useful tool in the clinical assessment of cognitive changes [24]. The CDT is a well-tolerated and accepted test completed by the participant [25].

#### Physical activity record (T1, T2)

The participant states the number and duration of walks during the past 14 days and whether she/he walked together with a volunteer or somebody else.

#### Falls questionnaire (T1, T2)

This questionnaire, answered by the participant, serves to find out whether she/he experienced any falls during the intervention. The participant states whether falls required medical attention, what kind of treatment was needed and by whom it was provided.

#### Treated falls (T1, T2)

This questionnaire documents, whether the participant experienced any medically treated falls during the intervention. The medical staff states the date and diagnosis and whether the fall occurred during the intervention.

### Outcomes

We expect the intervention to impact upon muscle strength, balance and ability to walk. We therefore chose the Short Physical Performance Battery as the main outcome of the study. SPPB scores have been shown to be associated with disability in mobility and activities of daily living [26–28], future hospitalisation [29], health improvement [30] and mortality [30–32]. For these simple measures of performance, the SPPB shows a good reliability and validity [27].

As secondary outcomes we will evaluate quality of life (EQ-5D-5L), fear of falling (FES-I), physical activity (activity diary), cognitive executive function (clock drawing test), falls requiring medical attention, hospitalisation and death.

### Nested qualitative sub-study

We will invite selected volunteers to focus group meetings and participants to individual interviews. Using an interview guideline, they will be asked to discuss their experiences with respect to positive aspects and shortcomings of the intervention. A special focus will be on effects of the intervention on physical and psychosocial wellbeing and quality of life as well as specific challenges encountered by either volunteers or participants. All interviews will be audiotaped and transcribed verbatim. The resulting text will be analysed qualitatively by means of a text analysis.

### Statistical analysis

#### Sample size

Sample size calculation was carried out based on expected effects regarding the primary outcome (physical function measured by the Short Physical Performance Battery [13] after 6 months of intervention (T1). A change of 1 point has been shown to be clinically relevant (e.g. predictive of future hospitalisation, health improvement and mortality) [33]. The standard deviation in comparable samples has ranged between 2.6 and 2.8 points [30, 31]. With a conservative approach, we assumed the higher standard deviation (2.8) for our calculation. In an analysis of covariance (including baseline values of the primary outcome), with an R-square of 0.5 for the covariate and a power of 95% (1-β), sample sizes of *n*=103 will be required for each of the two groups. The total sample of 206 participants achieves 95% power to detect differences among the means using an F test with a 5% significance level. The size of the variation in the means is represented by their standard deviation, which is 0.50. The common standard deviation within a group is assumed to be 2.8.

In our pilot study [12] conducted in nursing homes, we experienced a loss of 28% during the 6 months intervention period mainly due to death and hospitalisation. From our experience, the loss of participants recruited in primary care practices will be lower. A British primary care and community based study, which examined a complex nurse-delivered intervention with self-paced exercises and therefore people who are likely to be physically healthier (compared to our participants), experienced a loss of 6% during 3 months [9]. Considering that we are now also recruiting participants in community settings (living at home, primary care) and following a conservative approach, we set the expected loss during the intervention period to 40% (mainly due to death and hospitalisation). Assuming a loss of 40% during the 6 months of intervention we will need a total sample of *n*=345 to achieve adequate power of 95% at our primary endpoint. This will also enable us to achieve enough power for the analyses of secondary endpoints. The study centre Marburg will aim to recruit 230 persons from the community setting and the study centre Witten 115 from nursing homes.

#### Data analysis

At T1, we will apply an analysis of covariance of the primary outcome physical function (SPPB scores). This outcome will be used to analyse the difference between the intervention and control group. In case of non-normally distributed data, we will apply a nonparametric covariance model [34]. The secondary outcomes will be evaluated with ANCOVAs, t-tests or distribution independent Mann-Whitney U-Tests. To compare the number of deaths, events leading to discontinuation of the intervention, falls requiring medical attention, and hospital treatments, we will either calculate a χ2-test, Fisher exact test for binary variables or conduct a log-rank-test. Since the number of falls or falling in general has shown to depend on fear of falling and the amount of physical activity, we will perform a mediator analysis to correctly identify the effect of our intervention on falling [35].

Furthermore, we will perform exploratory analyses (multifactorial ANCOVAs and in case of non-normally distributed data adjusted rank transform tests [36]) and dichotomise and evaluate whether the participants in the subgroups setting (community setting, nursing home), sex, rural/urban setting, frailty, and baseline SPPB score show different results in our primary outcome SPPB or in secondary outcomes. We will perform this analysis at T1 and T2.

The evaluation of the intervention will be performed as intention-to-treat (ITT). The ITT analysis will include all randomised participants irrespective of the actual performance of the intervention. In case a participant decides to withdraw consent, we will ask for a final evaluation.

#### Missing data

If the T1 or T2 evaluation of a participant cannot be performed, the research assistant will decide whether an informative (death, admission) or non-informative event (e.g. moving to another municipality) has caused the evaluation to be missed. Information on reasons for missing the T1 or T2 evaluation will be obtained from participants’ families, general practitioners or nursing teams.

Depending on this categorisation, the following methods for analysis taking into account missing values will be used: 1) informative missing: not able to attend [examination] because of previous nursing home admission, hospital admission or death - tied worst rank score method will be assigned a SPPB Score = 0. For secondary outcomes, we will proceed in a similar fashion. In the analysis, we will apply the worst-rank score analysis [37]. It considers only the relative ordering of the values and applies the minimum variance linear or best linear estimator. For secondary outcomes, we will proceed in a similar fashion. 2) In case of non-informative missing (not able to attend because participant moved to another place without one of the conditions mentioned above), we will proceed with multiple imputation. In case of non-normally distributed data, we apply a nearest neighbour multiple imputation approach to estimate the proportion of each then categorised variable [38]. For secondary outcomes, we will proceed in a similar fashion.

We will also perform an additional per-protocol analysis, which will only include participants whose follow–up examination has taken place at the designated time (6 months after baseline or follow-up ± 2 weeks). Individuals of the intervention group participating in less than 25% of the scheduled walks will be excluded in the per-protocol analysis.

## Discussion

This current paper presents the study protocol of a randomised, controlled interventional superiority study: the POWER study. This project targets older people with inadequate activity levels. With this study, we will evaluate a volunteer-supported physical activity intervention and expect this intervention to improve the overall health status of the participants in a wide range of health-related outcomes.

This is a pragmatic study evaluating the effect of an intervention consisting of three components: 1) motivation by another person; 2) physical assistance; 3) psycho-social support (company). The control treatment does not address these mechanisms directly but aims to keep participants interested and motivated to comply with study procedures.

This study includes some limitations. Despite the fact that participants are recruited through a range of different settings, there is a possibility of selection bias, as participants have to be willing to complete the intervention. Participants who do not participate may be more isolated, less active and have more health issues than the participants included in the study. Furthermore, selective attrition due to death and geographical mobility of participants is possible. As mentioned above, blinding of participants and the research team is impossible to achieve.

We propose and evaluate a simple, personal non-educational intervention that is addressing the problem of physical activity in the group of already physically restricted older people with a lack of social support. In our opinion, it is well suited for a wide range of individuals with widely differing educational and/or socioeconomic backgrounds. In addition, participants with language barriers, who would be left out of many educational programmes, will be able to participate in the same way as other participants. The intervention provides a realistic way to motivate older people to become physically more active. Not relying on professional input, it requires limited financial or staff resources. The fact that the service will be provided personally by volunteers, will increase participants’ motivation and improve their quality of life.

## Supplementary Information

**Additional file 1.** TIDieR Checklist.

## Data Availability

Not applicable.

## Abbreviations

BMBF: Federal Ministry of Education and Research
CDT: Clock-drawing-test
CFS: Clinical Frailty Scale
DLR: The German Aerospace Centre
FES-I: Falls Efficacy Scale
ICD: International Classification of Diseases
ITT: Intention-to-treat
KKS: Coordination Centre for Clinical Trials
MMSE: Mini-Mental-Status-Examination
SPPB: Short Physical Performance Battery
WHO: World Health Organization
